# Surgical experience and identification of errors in laparoscopic cholecystectomy

**DOI:** 10.1093/bjs/znad256

**Published:** 2023-08-23

**Authors:** Gemma L Humm, Adam Peckham-Cooper, Jessica Chang, Roland Fernandes, Naim Fakih Gomez, Helen Mohan, Deirdre Nally, Anthony J Thaventhiran, Roxanna Zakeri, Anaya Gupte, James Crosbie, Christopher Wood, Khaled Dawas, Danail Stoyanov, Laurence B Lovat

**Affiliations:** Wellcome/Engineering and Physical Sciences Research Council Centre for Interventional and Surgical Sciences, University College London, London, UK; UCL Division of Surgery and Interventional Science, University College London, London, UK; Leeds Institute of Emergency General Surgery, Leeds Teaching Hospital NHS Trust, Leeds, UK; Department of General Surgery, Shrewsbury and Telford Hospital NHS Trust, Royal Shrewsbury Hospital, Shrewsbury, UK; Department of General Surgery, East Kent Hospitals University Foundation Trust, William Harvey Hospital, Ashford, UK; UCL Division of Surgery and Interventional Science, University College London, London, UK; Department of Surgery, Peter MacCallum Cancer Centre, Melbourne, Victoria, Australia; Department of Surgery, University of Melbourne, Melbourne, Victoria, Australia; Department of General Surgery, Mater Misericordiae University Hospital, Dublin, Ireland; Department of General Surgery, Royal London Hospital, Barts Health NHS, London, UK; UCL Division of Surgery and Interventional Science, University College London, London, UK; Department of General Surgery, University College London Hospital NHS Foundation Trust, University College Hospital, London, UK; UCL Division of Surgery and Interventional Science, University College London, London, UK; UCL Division of Surgery and Interventional Science, University College London, London, UK; UCL Division of Surgery and Interventional Science, University College London, London, UK; Wellcome/Engineering and Physical Sciences Research Council Centre for Interventional and Surgical Sciences, University College London, London, UK; Wellcome/Engineering and Physical Sciences Research Council Centre for Interventional and Surgical Sciences, University College London, London, UK; UCL Division of Surgery and Interventional Science, University College London, London, UK

## Abstract

**Background:**

Surgical errors are acts or omissions resulting in negative consequences and/or increased operating time. This study describes surgeon-reported errors in laparoscopic cholecystectomy.

**Methods:**

Intraoperative videos were uploaded and annotated on Touch Surgery^TM^ Enterprise. Participants evaluated videos for severity using a 10-point intraoperative cholecystitis grading score, and errors using Observational Clinical Human Reliability Assessment, which includes skill, consequence, and mechanism classifications.

**Results:**

Nine videos were assessed by 8 participants (3 junior (specialist trainee (ST) 3–5), 2 senior trainees (ST6–8), and 3 consultants). Participants identified 550 errors. Positive relationships were seen between total operating time and error count (*r*^2^ = 0.284, *P* < 0.001), intraoperative grade score and error count (*r*^2^ = 0.578, *P* = 0.001), and intraoperative grade score and total operating time (*r*^2^ = 0.157, *P* < 0.001). Error counts differed significantly across intraoperative phases (H(6) = 47.06, *P* < 0.001), most frequently at dissection of the hepatocystic triangle (total 282; median 33.5 (i.q.r. 23.5–47.8, range 15–63)), ligation/division of cystic structures (total 124; median 13.5 (i.q.r. 12–19.3, range 10–26)), and gallbladder dissection (total 117; median 14.5 (i.q.r. 10.3–18.8, range 6–26)). There were no significant differences in error counts between juniors, seniors, and consultants (H(2) = 0.03, *P* = 0.987). Errors were classified differently. For dissection of the hepatocystic triangle, thermal injuries (50 in total) were frequently classified as executional, consequential errors; trainees classified thermal injuries as step done with excessive force, speed, depth, distance, time or rotation (29 out of 50), whereas consultants classified them as incorrect orientation (6 out of 50). For ligation/division of cystic structures, inappropriate clipping (60 errors in total), procedural errors were reported by junior trainees (6 out of 60), but not consultants. For gallbladder dissection, inappropriate dissection (20 errors in total) was reported in incorrect planes by consultants and seniors (6 out of 20), but not by juniors. Poor economy of movement (11 errors in total) was reported more by consultants (8 out of 11) than trainees (3 out of 11).

**Conclusion:**

This study suggests that surgical experience influences error interpretation, but the benefits for surgical training are currently unclear.

## Teaser

Surgical experience influences the identification and classification of surgical errors in laparoscopic cholecystectomy. Future research in error interpretation is required to explain these differences and how this may support surgical training.

## Introduction

Laparoscopic cholecystectomy is the standard of care for symptomatic gallstone disease, with over 60 000 procedures performed annually in the UK^1^. Laparoscopic cholecystectomy has recognized complications. These include iatrogenic injury, bile leak, and common bile duct (CBD) injury^2^, which carry health service costs^3^, patient morbidity and mortality^4^, and may lead to litigation^5,6^. Technical errors are risks of surgery; however, appropriate training and technical proficiency reduces errors and improves quality in laparoscopic surgery^7,8^. Standardization of surgical technique in laparoscopic cholecystectomy with consistent identification of the critical view of safety (CVS) has been well described^9–14^, and has been documented to reduce the risk of CBD injury^15–17^.

Observational Clinical Human Reliability Assessment (OCHRA) has been applied to laparoscopic cholecystectomy in simulation and in the operating theatre to assess the performance of both surgical trainees and consultant surgeons^9–14^. OCHRA classifies surgical errors by skill, consequence, and mechanism. An error is an act or omission of a procedure or execution, reflecting cognitive or technical skills^12^. Errors can be consequential, resulting in a negative consequence or an increase in duration of the surgical process necessitating a corrective action^12^. Inconsequential errors increase the likelihood of a negative consequence that under different circumstances could have a consequential effect^12^. OCHRA further classifies surgical errors using external error modes, which describe the mechanism by which the error occurred and has excellent inter-rater reliability (IRR) between experts. It can therefore be used to provide discriminative feedback^9–14^.

There are few data pertaining to surgical trainees’ perceptions of surgical error. One study^18^ using the Global Operative Assessment of Laparoscopic Skills (GOALS) found that consultant surgeons working outside of a research setting scored surgical trainee videos differently from those within the research setting. Understanding differences in perception of surgical errors could support surgeons’ and trainees’ reflections and actions following errors, and improve technical and non-technical skills training. This pilot study aimed to describe the errors identified in laparoscopic cholecystectomy videos, using OCHRA, by a sample of surgeons with different levels of experience.

## Methods

### Data set

The Cholec80 data set^19^ is a fully anonymized video data set of 80 laparoscopic cholecystectomies performed by 80 surgeons and includes intraoperative phase labels. Videos were selected randomly from the data set and uploaded to Touch Surgery^TM^ Enterprise (Digital Surgery Ltd, London (Medtronic)3), a combined software and hardware solution for securely recording, storing, and analysing surgical videos. Intraoperative phases annotated in this data set are: preparation and exposure, dissection of hepatocystic triangle, ligation and division of cystic structures, gallbladder dissection, gallbladder packaging, clean and coagulate, and gallbladder retrieval.

### Participant recruitment and training

General surgeons (surgical trainees at specialist trainee (ST) 3–8 level or equivalent, and consultants) in the UK and Ireland were invited to participate. Invitations were sent via training groups and hospital networks. Participants were instructed in the study design, methodology, and use of the annotation platform by online group meetings. Participants were given a previous laparoscopic OCHRA study^12^ to read, and, during online training, error definitions and error modes were described verbally and supported with video examples, with specific reference to the errors identified in previous research^12^. The training included a demonstration of error identification and labelling using the annotation platform. Participants had an opportunity to discuss the study and ask questions. The training session lasted for 2 h, and was followed by question and discussion time. Participants also received written instructions, which included step-by-step instructions on how to label videos with screenshots of the annotation platform. Participants were offered ongoing follow-up group and individual training sessions, as well as direct contact to answer any specific questions. Any technical issues were referred to the Digital Surgery (Medtronic) team. Participants were asked to watch each video, score, identify video clips of surgical errors, and classify these errors using the following methodologies.

### Laparoscopic cholecystectomy operative grading score

Laparoscopic cholecystectomy grading scores often require clinical, biochemical, and radiological data in addition to intraoperative findings, and have been used to predict the likelihood of conversion to open operation. A 10-point scoring system for interoperative grading of cholecystitis^20^ (*Table S1*) was used to grade technical difficulty. BMI was omitted from the present study owing to lack of clinical information. Total scores only were considered here.

### Observational Clinical Human Reliability Assessment

The definitions used in this study were those from a previous OCHRA study^12^ (*Table 1*).

**Table 1:** Definitions of errors and external error modes

Term	Definition
Consequential error	Action or omission that resulted in a negative consequence, or increased duration of surgical procedure by necessitating a corrective action, which fell outside of acceptable limits
Inconsequential error	Action or omission that increased likelihood of negative consequence and under slightly different circumstances could have had a consequential effect
Procedural errors	Errors that reflect cognitive skills (EEM1–EEM6)
Executional errors	Errors that reflect executional skills (EEM7–EEM10)
EEM1	Step not done
EEM2	Step partially completed
EEM3	Step repeated
EEM4	Second step done in addition
EEM5	Second step done instead of first step
EEM6	Step done out of sequence
EEM7	Step done with too much force, speed, depth, distance, time or rotation
EEM8	Step done with too little force, speed, depth, distance, time or rotation
EEM9	Step done in wrong orientation, direction, or point in space
EEM10	Step done on/with wrong object (or plane)

EEM, external error mode.

### Annotation platform and methodology

Participants used Touch Surgery^TM^ Enterprise to annotate surgical errors in 10 complete, unedited videos. Specific labels were developed for this study, which included an ‘error start’ and ‘error stop’ label for each of the 10 external error modes, as well as additional tags for consequential and inconsequential error classification. A free-text box allowed participants to describe the errors with reference to previous research. Videos could be played back and stopped and/or paused as required, and the software would highlight the labels during playback. Digital Surgery provided a .csv file with case, and error durations, classification labels, and free-text descriptions.

### Data extraction and statistical analysis

Participants were divided for analysis into surgical experience subgroups: juniors (ST3–5 or equivalent), seniors (ST6–8 or equivalent), and consultants. Free-text data were coded using the descriptions of errors from a previous study^12^ (*Tables S2* and *S3*).

All data were treated as non-normally distributed. Median (i.q.r., range) was calculated for error counts, and either the Friedman (χ^2^_F_(d.f.)) or Kruskal–Wallis (H(d.f.)) statistic was calculated to compare subgroups. For comparisons of continuous data, simple linear regression was used to determine relationships using GraphPad Prism^®^ 9 for MacOS^®^ version 9.4.1 (458) (GraphPad Software, Boston, MA, USA). The intraclass coefficient (ICC) was calculated using a two-way mixed-effects model with absolute agreement for the total intraoperative grade score using SPSS^®^ for MacOS^®^ version 29.0.0.0 (241) (IBM, Armonk, NY, USA).

## Results

### Participants and video data set

Some 13 surgeons volunteered, none of whom had previous experience of video annotation. Two participants left the study before annotation training and one after, so data from 10 participants were analysed. Consultants reported over 500 logged procedures. The reported experience of juniors and seniors was a median of 38.5 (range 24–55) and 156 (96–280) procedures respectively.

Nine videos were analysed; one was excluded because of incomplete recording. Mean(s.d.) duration was 43.31(26.27) min (*Table S4*). Inter-rater agreement was excellent in assessing the intraoperative grade score (total value) (ICC 0.909, 95 per cent c.i. 0.767 to 0.974; *P* < 0.001).

### Error counts

**Fig. 1:**
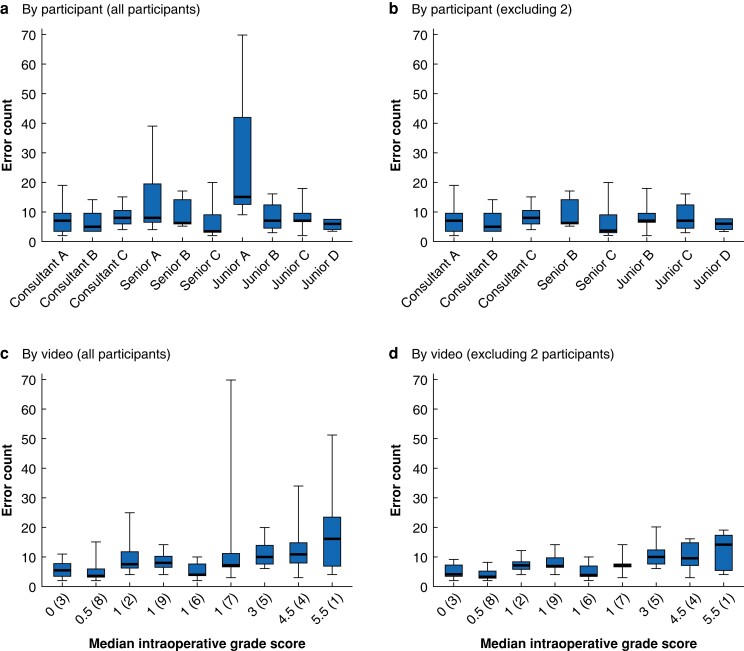
Box and whisker plots showing total error count by participant and by video Total cumulative error counts **a** by participant, all participants, **b** by participant, outlying two participants excluded, **c** by video, all participants, and **d** by video, outlying two participants excluded. In **c** and **d**, the *x*-axis label shows median intraoperative grade score by increasing score, with video number in parentheses. The highest operative grade score represents the most challenging case in the sample, and is positioned on the far right of the *x*-axis. Median values (bold line) i.q.r. (box), and range (error bars) are shown. **a** χ^2^_F_(9) = 28.39, *P* < 0.001, **b** χ^2^_F_(7) = 6.88, *P* = 0.442, **c** χ^2^_F_(8) = 32.20, *P* < 0.001, **d** χ^2^_F_(8) = 23.63, *P* = 0.003 (Kruskal Wallis/Friedman test).

Some 907 errors were reported. Junior A and senior A were outlying participants in videos 7 and 1 respectively. *Figure 1* shows error counts, including the change in significance in median error counts when outlying participants were excluded, suggesting that senior A and junior A over-reported errors. After their exclusion, 550 errors were analysed and classified by skill and further consequence in approximate ratios of 1 : 2, and 8 : 1 and 6 : 4 respectively (*Fig. S1*).

**Fig. 2:**
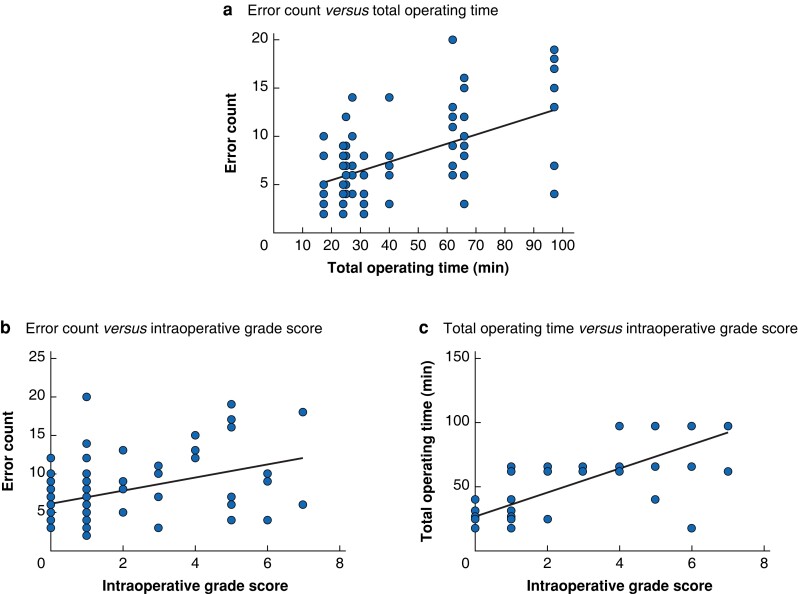
Scatter plots and linear regression analysis of relationships between total operating time, total error count, and intraoperative grade score **a** Total operating time *versus* error total count, **b** intraoperative grade score *versus* total error count, and **c** intraoperative grade score *versus* total operating time. **a***r*^2^ = 0.284, *P* < 0.001, **b***r*^2^ = 0.578, *P* = 0.001, **c***r*^2^ = 0.157, *P* < 0.001.

**Fig. 3:**
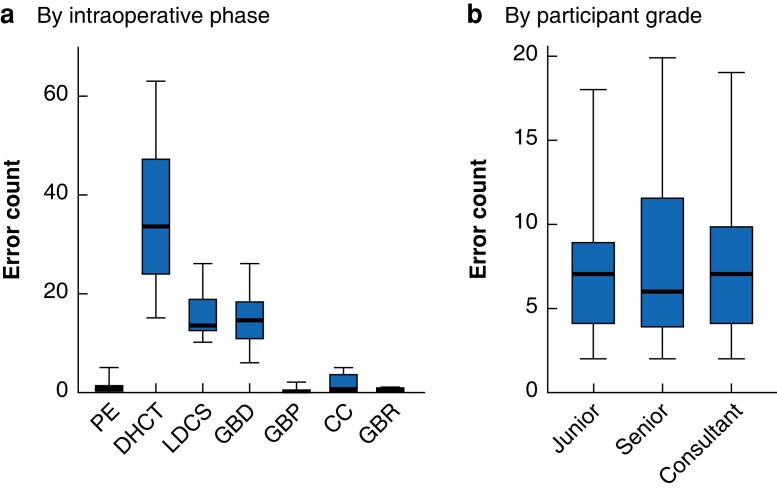
Box and whisker plot showing total cumulative error counts by intraoperative phase and surgical experience Total cumulative error count **a** by intraoperative phase and **b** participant grade. Median values (bold line) i.q.r. (box), and range (error bars) are shown. PE, preparation and exposure; DHCT, dissection of hepatocystic triangle; LDCS, ligation and division of cystic structures; GBD, gallbladder dissection; GBP, gallbladder packing; CC, clean and coagulate; GBR, gallbladder retrieval. **a** H(6) = 47.06, *P* < 0.001, **b** H(2) = 0.03, *P* = 0.987 (Kruskal–Wallis test).

Linear regression analysis showed positive linear relationships between total operating time and error count (*Fig. 2a*), intraoperative grade score and error count (*Fig. 2b*), and intraoperative grade score and total operating time (*Fig. 3c*).

### Video timeline analysis

Error timelines identified consistent error detection across several participants (*Figs. S2–S10*).

### Intraoperative phase analysis

A significant difference was found in median error counts across seven phases (H(6) = 47.06, *P* < 0.001). Errors were frequently reported in dissection of hepatocystic triangle (total 282; median 33.5 (range 15–63)), ligation/division of cystic structures (total 124, median 13.5; range 10–26), and gallbladder dissection (total 117; median 14.5 (range 6–26)). Errors were reported infrequently in the remaining intraoperative phases (*Fig. 3a*).

### Surgical experience subgroup analysis

**Fig. 4:**
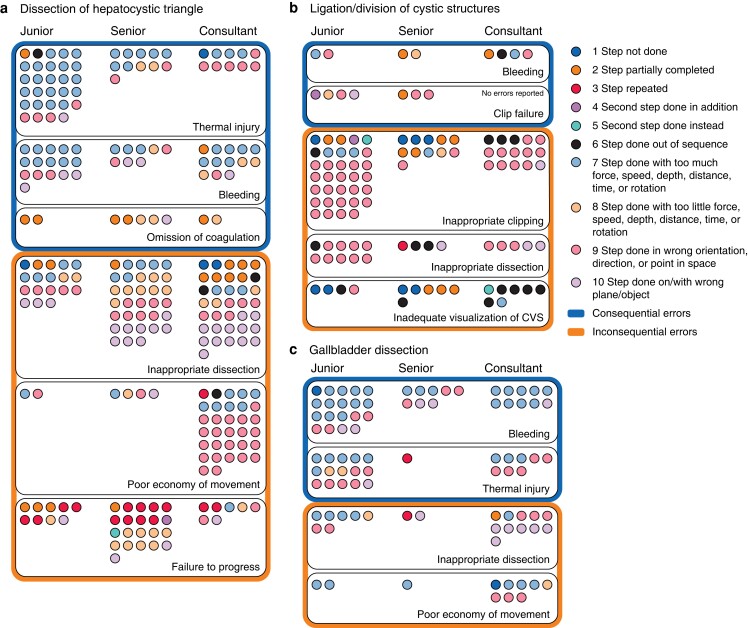
Reported consequential and inconsequential external error mode in relation to surgical experience **a** Dissection of hepatocystic triangle, **b** ligation/division of cystic structures, and **c** gallbladder dissection. CVS, critical view of safety.

Participants’ error counts were analysed in surgical experience subgroups. No significant difference in median error counts was found between subgroups (H(2) = 0.027, *P* = 0.987) (*Fig. 3b*). Reported errors matched with external error modes by each subgroup are shown for dissection of hepatocystic triangle, ligation/division of cystic structures, and gallbladder dissection in *Fig. 4*.

#### Dissection of hepatocystic triangle

In all, 282 errors were reported. Frequently reported consequential errors were thermal injury, bleeding, and omission of coagulation. Frequently reported inconsequential errors were inappropriate dissection, poor economy of movement, and failure to progress. All grades reported thermal injuries, mostly classified as executional errors. Trainees (juniors and seniors) mostly classified these errors as excessive force, speed, depth, distance, time or rotation. Consultants mostly classified these errors as energy applied in the incorrect direction/orientation.

All grades reported bleeding, mostly by excessive force, speed, depth, distance, time or rotation. Omitting coagulation was mainly classified as a step partially completed. Senior surgical trainees and consultants additionally classified errors as insufficient energy for coagulation. Inappropriate dissection was classified by consultants and seniors as dissection in the wrong plane, whereas trainees classified more of these errors as excessive force, speed, depth, distance, time or rotation, and poorly directed dissection. Seniors reported more dissection errors as insufficient force, speed, depth, distance, time or rotation. Inappropriate dissection as a procedural error was reported as step not performed and incomplete dissection. Poor economy of movement was reported by consultants considerably more than trainees, and classified as poorly directed instruments. Failure to progress was reported by seniors more than juniors and consultants, and classified mostly as a procedural error. Failure to progress was classified by seniors as an executional error, and as a poorly directed action and as action in the incorrect plane by consultants.

#### Ligation/division of cystic structures

Some 124 errors were reported. Frequently reported consequential errors were bleeding and clip failure Frequently reported inconsequential errors were inappropriate clipping, inappropriate dissection, and inadequate visualization of the CVS. Bleeding was reported by all grades and mostly classified as an executional error. Clip failure was reported only by trainees, and mainly classified as executional.

Juniors reported more inappropriate clipping than senior trainees and consultants, and classified it as clip applicators deployed in the incorrect orientation with clipper tips not visualized. Procedural errors concerned the number or sequence of clip application. Seniors showed variation in classification of skill, whereas consultants only reported clip applicators deployed in the incorrect orientation. Inappropriate dissection was classified as an executional error, including poorly directed division of cystic structures and dissection in the incorrect plane. This was reflected across grades. Procedural errors were fewer, with repetition of step and errors of sequence reported. Inadequate visualization of the CVS was reported by all grades, and was mostly classified as a procedural error. Consultants reported errors of sequence more frequently than trainees, whereas trainees reported failure to achieve CVS and incomplete dissection of the hepatocystic triangle. The reported executional errors concerned dissection in the incorrect direction and with excessive force, speed, depth, distance, time or rotation. This was reported by consultants only.

### Gallbladder dissection

A total of 117 errors were reported. Frequently reported consequential errors were bleeding, thermal injury, and omission of coagulation. Frequently reported inconsequential errors were inappropriate dissection, poor economy of movement, and failure to progress. Bleeding was reported by all grades, and classified as excessive force, speed, depth, distance, time or rotation, dissection in the incorrect direction, and dissection in the incorrect plane. Thermal injury was reported by juniors and consultants mostly as diathermy in the incorrect direction, or excessive force, speed, depth, distance, time or rotation. Only juniors reported this error because of inadequate force, speed, depth, distance, time or rotation and diathermy in the incorrect plane, whereas seniors reported an additional use of diathermy. Inappropriate dissection was reported mostly by consultants as dissection in the incorrect plane; juniors did not identify this. Dissection in the incorrect direction and with inadequate force, speed, depth, distance, time or rotation was reported only by juniors. Procedural errors included a repeated step and partially completed step. Poor economy of movement was reported more by consultants than trainees. Consultants mainly classified this as movement of instruments with excessive force, speed, depth, distance, time or rotation and in the incorrect direction. There were few reports of inadequate force, speed, depth, distance, time or rotation and step partially completed.

### Focus group evaluation

Participants were invited to an online focus group to discuss their experience of the project; five attended with representation from all levels. All participants reported that the training and supporting materials were useful, and that regular contact was motivating. Participants noted that the explanation of OCHRA may be improved with example video clips to aid the understanding of definitions, and felt that their understanding and confidence improved with practice. Participants felt that some external error modes were irrelevant, but acknowledged this could be because these mechanisms were not identified. Participants reported that they found OCHRA subjective, and an observer bias could be present if a judgement had been made previously on the skill of the surgeon. Participants reported that they perceived an educational benefit in participating, including increased reflection on their own technical skills. Participants perceived merit in receiving feedback on errors with video coaching. One consultant reported recording their procedures as standard of care. Trainees reported they did not record their procedures as standard; however, this study reinforced the importance of critical review of surgical videos, both their own and those of peers. All participants were interested in participating in future studies.

## Discussion

No significant difference in error counts was found between surgical experience subgroups. However, this study showed variability in descriptions of some events and external error modes, suggesting disagreement on event occurrence. Nonetheless, when participants agreed on events there was consistency in error classification of skill, consequence, and mechanism.

The variable interpretation could reflect trainees’ journeys through the experiential learning cycle^21^ or the conscious-competence model^22^. Perhaps the ability to identify more subtle errors, with the apparent dismissal of more apparent errors, reflects a consultant’s unconscious competence^22^. Trainees, particularly juniors, may approach error classification with more caution than their seniors, and be more aware of some errors as a result of recent training and assessment. Consultants may disregard some minor consequential errors as sequelae are unlikely. Using their experience of training others may allow detection of more subtle errors, such as poor economy of movement. It is likely that the identification of errors is relevant to a trainee’s level of competence in laparoscopic cholecystectomy. Before ST3, trainees have assisted and learned the procedural steps at course granularity. Feedback on practical experience in laparoscopic cholecystectomy at ST3–6 focuses on the execution of surgical skills to safely dissect the hepatocystic triangle, identification of the CVS, and learning how to proceed safely with uncertain anatomy. Finally, ST7–8 trainees refine their decision-making for autonomous practice. A US study^23^ compared resident and attending operative decision-making in laparoscopic cholecystectomy, and found that attendings identified significantly more operative steps and cross-links than residents, demonstrating that attendings were analysing surgical processes at a finer granularity. This could explain the variability in interpretation of errors between groups in the present study, which is contrary to suggestions that the IRR in laparoscopic skills assessment is not inherent and deteriorates over time and without regular training^18^. Therefore, aiding reflection and analysis of one’s own performance, or the performance of others, could be beneficial in facilitating transition to the next level of competence.

Higher error counts were found in the operative phases dissection of hepatocystic triangle, ligation/division of cystic structures, and gallbladder dissection, with an approximate ratio of 2 : 1 for consequential : inconsequential errors. Executional, consequential errors tended to comprise use of instruments in the wrong direction, plane or with too much force, speed, depth, distance, time or rotation, and resulted in either thermal injuries to small vessels or liver bleeds. These findings are consistent with previous research^9,10,12,13^. This study suggests that challenging procedures, with longer total operating times, have higher error counts. However, the magnitude of association is small and was with variability in participants’ assessment of procedure severity.

This study has limitations. The data set is based on intraoperative videos, so it is not possible to comment on or understand the impact of patient factors, such as co-morbidity, or surgeon performance-shaping factors, including time available, stress/stressors, complexity, experience/training, procedures, ergonomics/human-machine interface, fitness for duty, and work processes^24^. Participants may have interpreted events differently if this information had been available.

This study does not provide information about missed, misinterpreted or unnoticed events, lenient assessment, or perceived irrelevance. Possibly, the application of OCHRA is too complex, or the interpretation of semantics results in inconsistencies. Asking participants to assess performance using an additional metric, such as GOALS, could have enriched the data, allowed comparison, and identified examples of good performance. This pilot study has a small sample size and few more challenging procedures, limiting the generalizability of the results. However, this is likely a reflection of the distribution in the general population and bias created by the surgical team publishing ‘better’ cases for scrutiny. The participants commented on the visibility of the CVS, which was not seen in previous studies. Participants were not instructed to comment on the CVS in either this study or the previous^12^. OCHRA does not provide a framework for assessing the CVS, but this should be considered in future work.

Larger samples of videos and participants over a longer interval may uncover a learning curve in error identification and labelling. Improving training materials, including more examples, could improve participant engagement and agreement, but could introduce bias towards the examples given. A prestudy trial and assessment of participants’ ability could have aided participants’ familiarity and may have avoided exclusions. It is unclear why the outlying participants overidentified errors. No participants had previous experience of video annotation or OCHRA. Perhaps they already had an interest in technology and education; this was not explored, but it is likely that most of those volunteering to participate in a technological or educational study would have such interests.

Laparoscopic surgery is a bimanual skill; opposing external error modes could have been misinterpreted. The laterality of instruments was not identified in previous studies and should be considered in future work. Coding of free-text descriptions could introduce observer bias, which could be reduced by providing categorical error descriptions for selection. Finally, this study has not considered the clinical sequelae of errors, importantly, to distinguish errors that reflect poor clinical outcomes or reflect techniques from an expert perspective. Prospective study designs with postoperative follow up are needed.

Future work could consider error classification as a structured method of feedback in surgical training and whether engaging in video labelling has its own educational benefit.

Video annotation is a valuable tool in the development of artificial intelligence systems, and future work should consider this. Achieving the volume of expert annotation data required can be a challenge, and may require crowdsourcing or large-scale collaborative research projects^25^.

To the authors’ knowledge, this is the first study of more than two raters using OCHRA in laparoscopic cholecystectomy to investigate variability in error classification between surgical experience subgroups. The analysis has shown that surgical experience influences the classification of surgical errors in laparoscopic cholecystectomy. Future research is needed into how this can affect feedback and how this may best support surgical training.

## Supplementary Material

znad256_Supplementary_Data

## Data Availability

The data sets generated during and/or analysed during the present study are available from the corresponding author on reasonable request.
