# Supplementary material for: Surgical experience and identification of errors in laparoscopic cholecystectomy
Source: Br J Surg. 2023 Aug 23;110(11):1535–42. doi: 10.1093/bjs/znad256 (PMC10564403; doi:10.1093/bjs/znad256)
Supplement: znad256_Supplementary_Data [file znad256_supplementary_data.docx]

# Title page

Surgical experience influences the identification of errors in laparoscopic surgery. A pilot study.

# Authors

Gemma L Humm^1,2^, Adam Peckham-Cooper^3^, Jessica Chang^4^, Roland Fernandes^5^, Naim Fakih Gomez^2^, Helen Mohan^6, 7^, Deirdre Nally^8^, Anthony J Thaventhiran^9^, Roxanna Zakeri^2^, Anaya Gupte^9^, James Crosbie^2^, Christopher Wood^2^, Khaled Dawas^2^, Danail Stoyanov^1^, Laurence B Lovat^1,2^.

# Affiliations

^1^ Wellcome/ Engineering and Physical Sciences Research Council Centre for Interventional and Surgical Sciences. University College London, Charles Bell House, 43-45 Foley Street, London, W1W 7TY, UK.

^2^ UCL Division of Surgery and Interventional Science, University College London, Charles Bell House, 43-45 Foley Street, London, W1W 7TY, UK.

^3^ Leeds Institute of Emergency General Surgery, Leeds Teaching Hospital NHS Trust, Great George Street, Leeds LS1 3EX, UK.

^4^ Department of General Surgery, The Shrewsbury and Telford Hospital NHS Trust, Royal Shrewsbury Hospital, Mytton Oak Road, Shrewsbury SY3 8XQ, UK.

^5^ Department of General Surgery East Kent Hospitals University Foundation Trust, William Harvey Hospital, Kennington Rd, Willesborough, Ashford TN24 0LZ, UK.

^6^ Department of Surgery, Peter MacCallum Cancer Centre, Melbourne Australia.

^7^ Department of Surgery, University of Melbourne.

^8^ Department of General Surgery Mater Misericordiae University Hospital, Eccles Street, Dublin 7, Ireland.

^9^ Department of General Surgery Royal London Hospital, Barts Health NHS, Whitechapel Road, Whitechapel, London E1 1FR, UK.

^9^ Department of General Surgery, University College London Hospital NHS Foundation Trust, University College Hospital 235 Euston Road, London. NW1 2BU, UK.

# Corresponding author

Miss Gemma Humm

Wellcome/ Engineering and Physical Sciences Research Council Centre for Interventional and Surgical Sciences. University College London, Charles Bell House, 43-45 Foley Street, London, W1W 7TY, UK.

g.humm@ucl.ac.uk

**Supplementary Materials - Index**

| Supplementary Figures and Tables |  |
| --- | --- |
| S1. Intraoperative grade score adapted from Sugrue *et al.(2015)*^21^. Body mass index was not available for this study. | *pag. 6* |
| S2. Inconsequential error codes for free text responses, adapted from Tang et.al (2004)^12^ | *pag. 7* |
| S3. Consequential error codes for free text responses, adapted from Tang et.al (2004)^12^ | *pag. 7* |
| S4. Sample video dataset case duration and intraoperative grade scores. | *pag. 7* |
| S5. Hierarchy diagram showing the skill and consequence classification of the total errors identified in the sample. | *pag. 8* |
| S6. Timeline of errors identified by each participant. Arrows mark where there is >70% agreement (6 or more participants) in error in video 1. | *pag. 8* |
| S7. Timeline of errors identified by each participant. Arrows mark where there is >70% agreement (6 or more participants) in error in video 2. | *pag. 8* |
| S8. Timeline of errors identified by each participant. Arrows mark where there is >70% agreement (6 or more participants) in error in video 3. | *pag. 8* |
| S9. Timeline of errors identified by each participant. Arrows mark where there is >70% agreement (6 or more participants) in error in video 4. | *pag. 8* |
| S10. Timeline of errors identified by each participant. Arrows mark where there is >70% agreement (6 or more participants) in error in video 5. | *pag. 8* |
| S11. Timeline of errors identified by each participant. Arrows mark where there is >70% agreement (6 or more participants) in error in video 6. | *pag. 8* |
| S12. Timeline of errors identified by each participant. Arrows mark where there is >70% agreement (6 or more participants) in error in video 7. | *pag. 8* |
| S13. Timeline of errors identified by each participant. Arrows mark where there is >70% agreement (6 or more participants) in error in video 8. | *pag. 8* |
| S14. Timeline of errors identified by each participant. Arrows mark where there is >70% agreement (6 or more participants) in error in video 9. | *pag. 8* |

| Description | Score |
| --- | --- |
| Adhesions <50% of gallbladder | 1 |
| Adhesions burying gallbladder | 3 |
|  | Maximum score 3 |
| Distension/contraction of gallbladder | 1 |
| Unable to grasp with atraumatic lap forceps | 1 |
| Stone >1cm impacted in Hartmann’s pouch | 1 |
| Access |  |
| Body Mass Index >30 | 1 |
| Adhesions from surgery limiting access | 1 |
| Severe Sepsis/Complications | 1 |
| Bile/pus outside of gallbladder | 1 |
| Time to identify cystic artery and duct >90mins | 1 |
| TOTAL (MAXIMUM 10) |  |

S1. Intraoperative grade score adapted from Sugrue *et al.(2015)*^21^. Body mass index was not available for this study.

| A | Overshoot of instrument | Poor economy of movement |
| --- | --- | --- |
| B | Non-visualisation of cutting tip | Unsafe dissection |
| C | Instrument out of endoscopic view /poor instrument control | Poor economy of movement |
| D | Energy applied without visualisation | Poor economy of movement |
| E | Cutting without lifting tissue from structures beneath | Unsafe dissection |
| F | Inappropriate dissection | Unsafe dissection |
| G | Insertion of instrument into the wrong plane | Unsafe dissection |
| H | Inappropriate tissue handling |  |
| I | Instrument in wrong direction | Poor economy of movement |
| J | Inappropriate clipping | Unsafe ligation |
| K | Avulsion of tissue | Inappropriate tissue handling |
| L | Failure to progress |  |
| M | Port reinsertion | Port management |
| N | Poor port placement | Port management |
| O | Critical View of Safety inadequately visualised | CVS |
| P | Inadequate camera view | Inadequate camera view |
| Q | Inadequate countertraction | Unsafe dissection |

S2. Inconsequential error codes for free text responses, adapted from Tang et.al (2004)^12^

| A | Perforation of gallbladder | Perforation |
| --- | --- | --- |
| B | Perforation of small bowel | Perforation |
| C | Bleeding – liver injury | Bleeding |
| D | Bleeding – small vessel | Bleeding |
| E | Bleeding – omental injury | Bleeding |
| F | Injury to cystic duct | N/A |
| G | Omitting to coagulate bleeding | Bleeding |
| H | Burn – liver | Thermal injury |
| I | Burn – Diaphragm | Thermal injury |
| J | Burn – duodenum | Thermal injury |
| K | Burn – abdominal wall | Thermal injury |
| L | Burn – gallbladder | Thermal injury |
| M | Instrument malfunction | Instrument malfunction |
| N | Replace clip | Ligation failure |
| O |  | Contamination |
| p | Clip dislodged | Ligation failure |

S3. Consequential error codes for free text responses, adapted from Tang et.al (2004)^12^

| New video number | Case duration (mins) | Median, IQR (range)intraoperative grading score |
| --- | --- | --- |
| 1 | 97 | 5.5, 7.75 (4-7) |
| 2 | 25.3 | 1, 1 (0-1) |
| 3 | 24.1 | 0, 0 (0-1) |
| 4 | 65.8 | 4.53 (1-6) |
| 5 | 61.9 | 3, 5 (0-6) |
| 6 | 17.3 | 1, 5.75 (0-6) |
| 7 | 40 | 1, 4 (0-5) |
| 8 | 31.2 | 0, 1 (0-1) |
| 9 | 27.2 | 1, 1 (0-1) |

S4. Sample video dataset case duration and intraoperative grade scores.


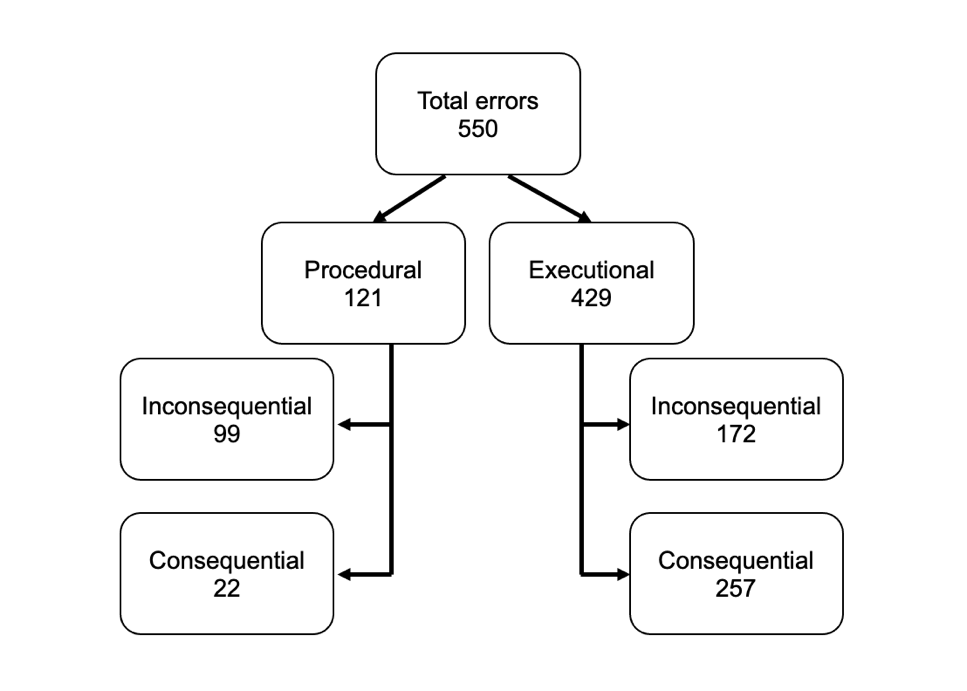


S5. Hierarchy diagram showing the skill and consequence classification of the total errors identified in the sample.


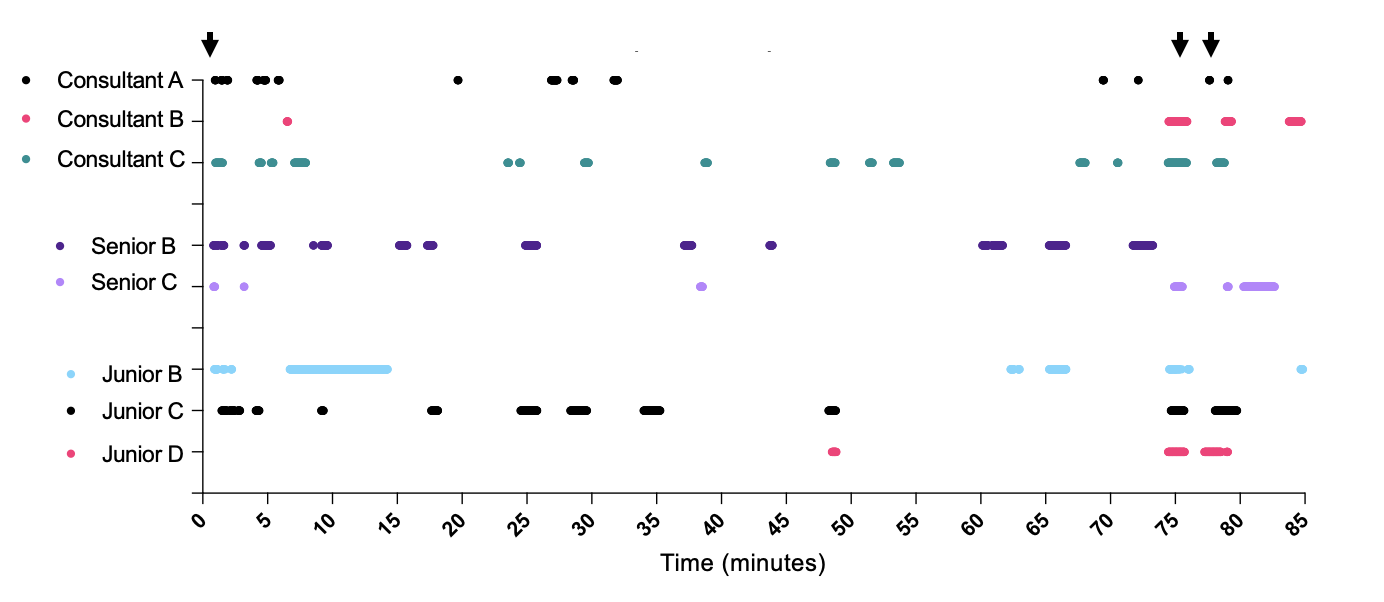


S6. Timeline of errors identified by each participant. Arrows mark where there is >70% agreement (6 or more participants) in error in video 1.


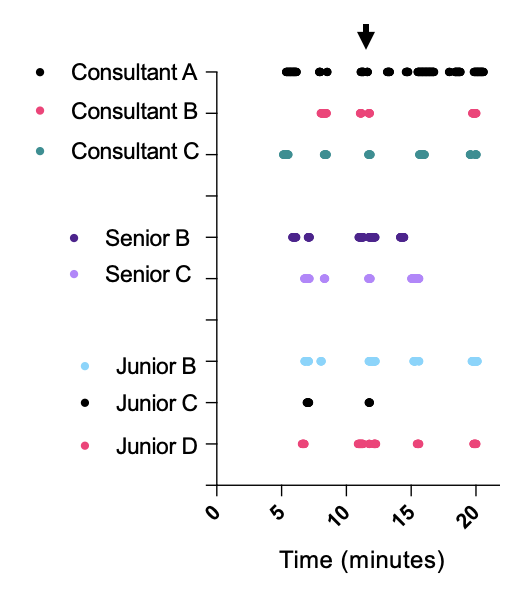


S7. Timeline of errors identified by each participant. Arrows mark where there is >70% agreement (6 or more participants) in error in video 2.


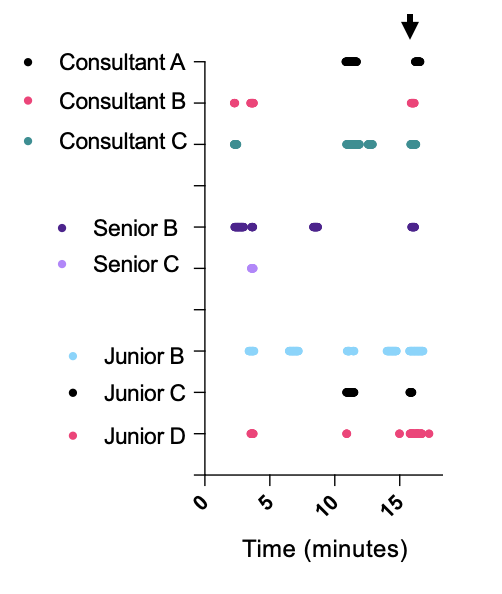


S8. Timeline of errors identified by each participant. Arrows mark where there is >70% agreement (6 or more participants) in error in video 3.


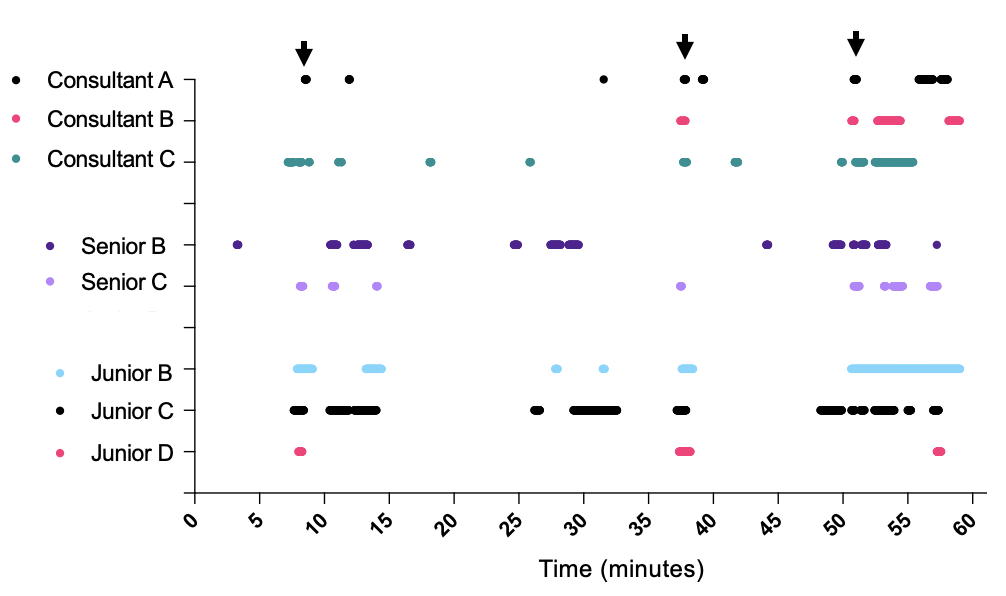


S9. Timeline of errors identified by each participant. Arrows mark where there is >70% agreement (6 or more participants) in error in video 4.


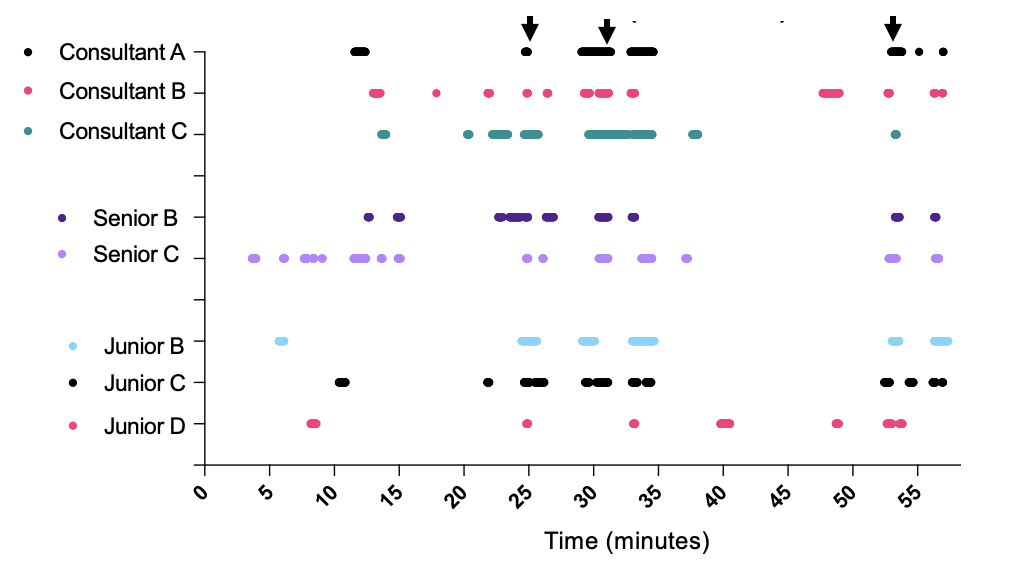


S10. Timeline of errors identified by each participant. Arrows mark where there is >70% agreement (6 or more participants) in error in video 5.


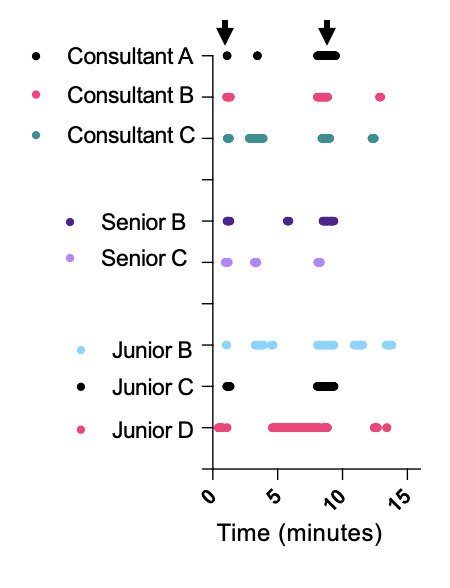


S11. Timeline of errors identified by each participant. Arrows mark where there is >70% agreement (6 or more participants) in error in video 6.


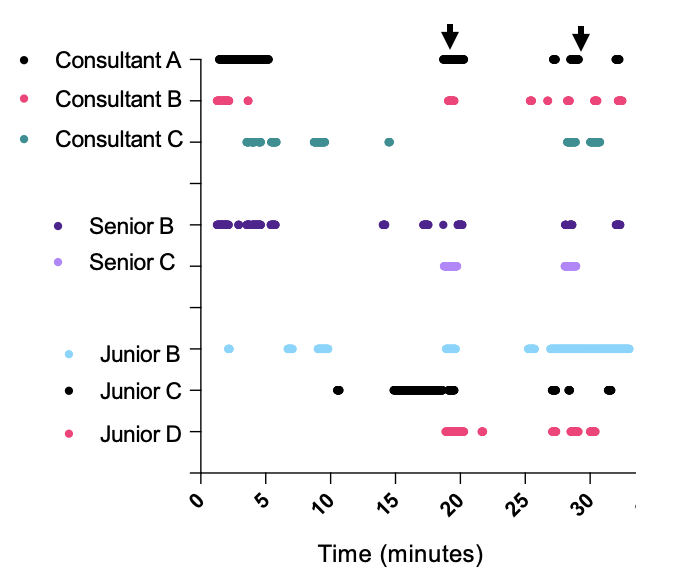


S12. Timeline of errors identified by each participant. Arrows mark where there is >70% agreement (6 or more participants) in error in video 7.


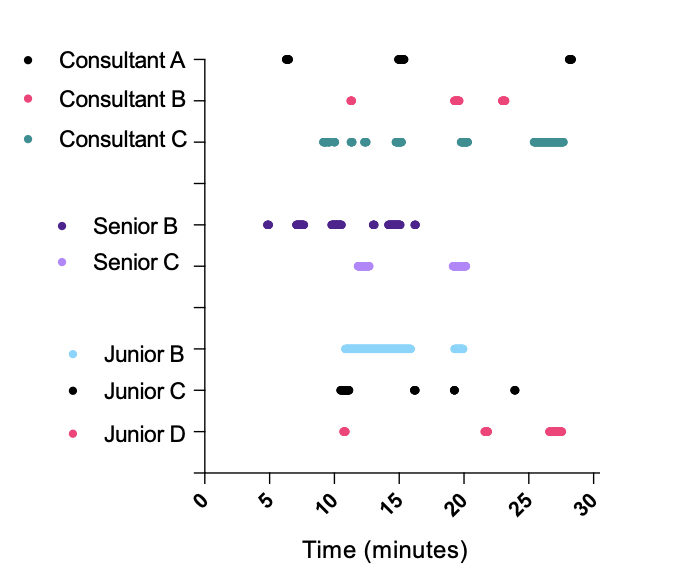


S13. Timeline of errors identified by each participant. Arrows mark where there is >70% agreement (6 or more participants) in error in video 8.


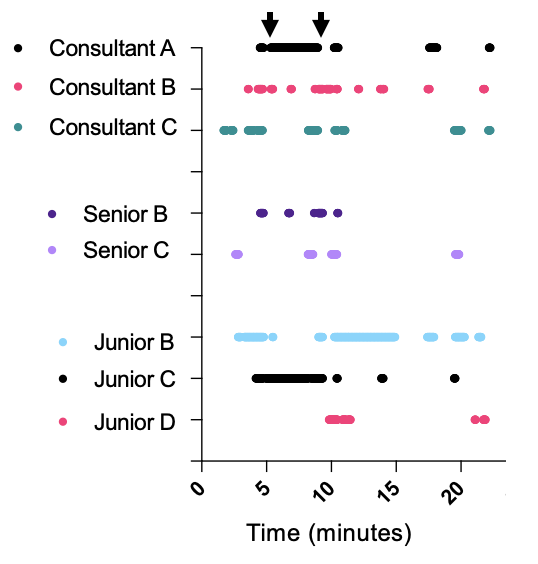


S14. Timeline of errors identified by each participant. Arrows mark where there is >70% agreement (6 or more participants) in error in video 9.
